# Infections with the SARS-CoV-2 Delta variant exhibit fourfold increased viral loads in the upper airways compared to Alpha or non-variants of concern

**DOI:** 10.1038/s41598-022-18279-5

**Published:** 2022-08-17

**Authors:** Christian J. H. von Wintersdorff, Jozef Dingemans, Lieke B. van Alphen, Petra F. G. Wolffs, Brian M. J. W. van der Veer, Christian J. P. A. Hoebe, Paul H. M. Savelkoul

**Affiliations:** 1 Department of Medical Microbiology, Care and Public Health Research Institute (CAPHRI), Maastricht University Medical Center+ (MUMC+), P. Debyelaan 25, 6229 HX Maastricht, The Netherlands; 2 Department of Sexual Health, Infectious Diseases and Environment, South Limburg Public Health Service, Heerlen, The Netherlands

**Keywords:** SARS-CoV-2, Public health

## Abstract

There has been a growing body of evidence that the severe acute respiratory syndrome coronavirus 2 (SARS-CoV-2) Delta variant (B.1.617.2) shows enhanced transmissibility and increased viral loads compared to other variants. A recent study has even suggested that respiratory samples from people infected with the Delta variant can harbor up to 1000 times higher viral loads compared to samples with variants that are more closely related to the original Wuhan strain, although the sample size of this study (n = 125) was very limited. Here, we have compared the viral load in 16,185 samples that were obtained in periods during which non-VOC, the Alpha (B.1.1.7) or Delta variant (B.1.617.2) were dominant as evidenced by genomic surveillance. We found that the Delta variant contained about fourfold higher viral loads across all age groups compared to the non-VOC or Alpha variants, which is significantly lower than reported earlier. Interestingly, the increased viral load for the Delta variant seemed to be age-dependent, regardless of sex, as the viral load was about 14-fold higher for Delta compared to the non-VOC or Alpha variant in age group 0–20 years and fourfold higher in age group 21–40 years, while there was no difference in viral load between variants in age groups 41–60 and 61+ years, most likely as a consequence of a higher degree of vaccination in the older age groups.

## Introduction

During the ongoing COVID-19 pandemic, various variants of SARS-CoV-2 have emerged. Some of these variants have been labelled as variants of interest (VOI) or variants of concern (VOC) by the WHO due to their potentially increased transmissibility, disease severity or immune escape characteristics^1^. The B.1.1.7 or Alpha variant was shown to have a greatly increased transmissibility^2^ and rapidly became the dominant circulating SARS-CoV-2 variant in most countries including The Netherlands. In late 2020, the B.1.617.2 or Delta variant was identified and raised concern as it appeared to be even more highly transmissible than the Alpha variant^3–5^ and is also linked to possible immune escape^6–8^. Subsequently, the Delta variant rapidly became the new dominant SARS-CoV-2 variant throughout the world.

As the Delta variant established itself as the new dominant SARS-CoV-2 variant in The Netherlands, a seemingly increase in detected viral loads was noticed during routine diagnostic testing at our department. Increased viral loads in infected persons could potentially contribute to the increased transmissibility of the Delta variant. In this study, we investigated the link between viral loads of the Delta and other SARS-CoV-2 variants in infected persons.

## Results

### Viral load comparison based on variant-dominant time periods

**Figure 1:**
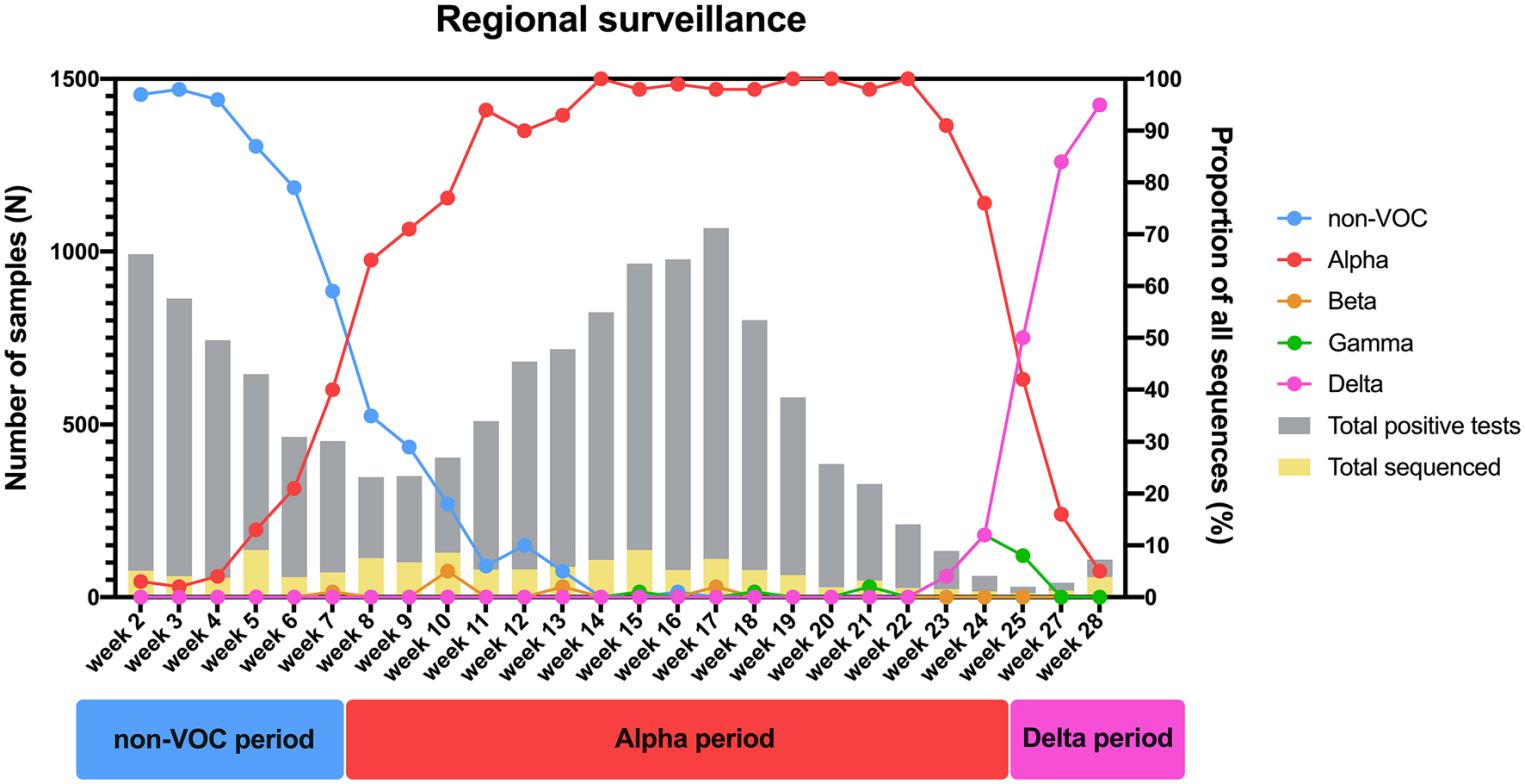
Distribution of circulating variants in the South Limburg region in time. The period during which a particular variant was dominant (> 50% of sequenced samples) is indicated below. Lines represent the relative proportion of each variant, whereas bars represent the total number of samples that were tested positive (grey) or were sequenced (yellow) in function of time. At least 7% of positive samples were sequenced per week.

In the first comparison, three distinct time periods were determined in which certain SARS-CoV-2 variants were dominant in our testing population in South-Limburg, the Netherlands. These periods were labelled as the non-VOC period (December 2020–February 2021), Alpha period (March 2021–June 2021) and Delta period (July 2021). The prevalence of SARS-CoV-2 variants in these time periods was based on whole-genome sequencing (WGS) data of our genomic surveillance program (Fig. 1), which is part of the Dutch national SARS-CoV-2 surveillance program.

**Figure 2:**
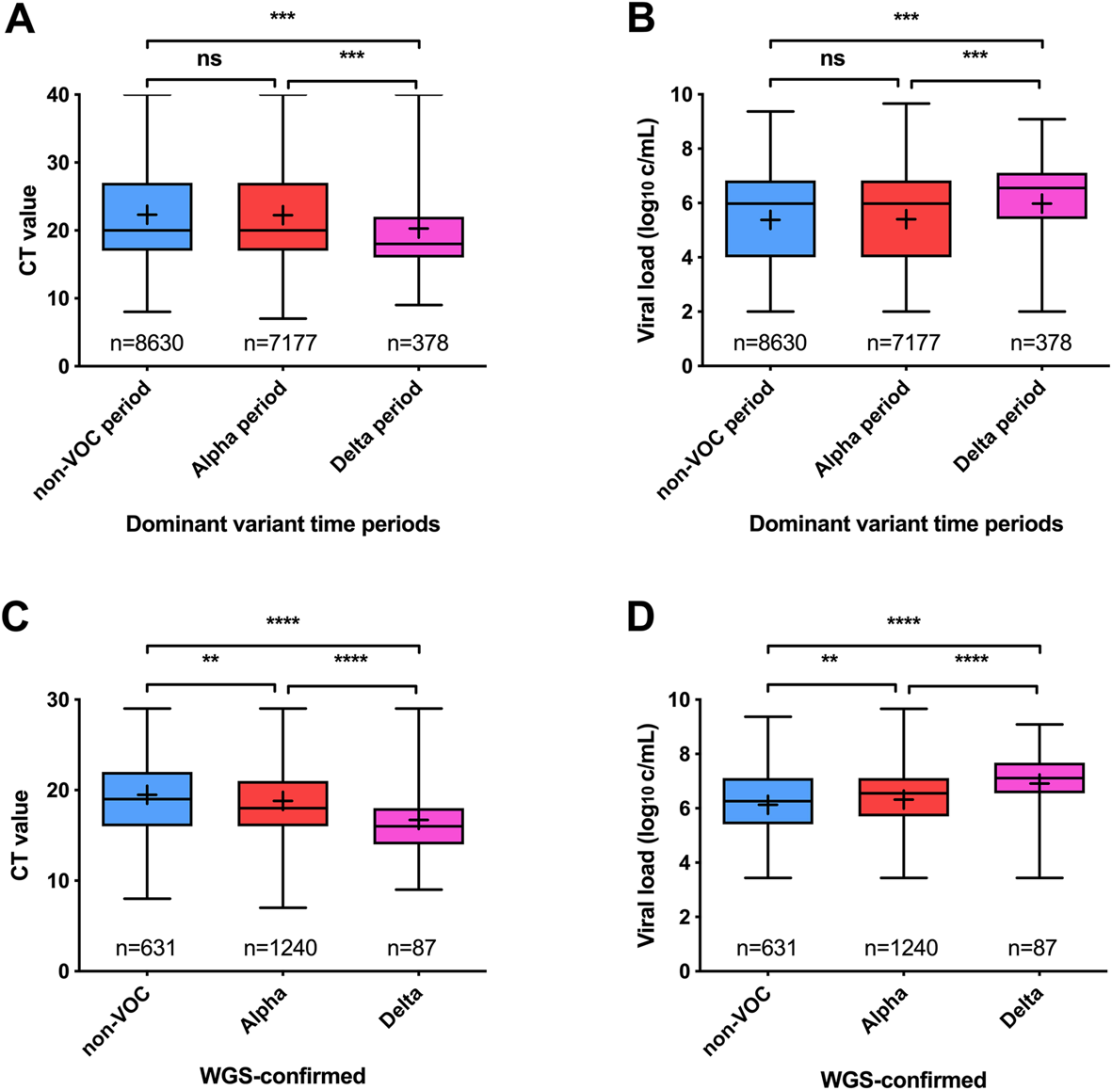
CT values (**A**) and viral loads (**B**) per time period dominated by non-VOC (Dec 2020–Feb 2020), Alpha (Mar 2020–Jun 2020) and Delta (Jul 2020) SARS-CoV-2 variants. CT values (**C**) and viral loads (**D**) per WGS confirmed SARS-CoV-2 variants. *Ns* not significant, **p < 0.01, ***P < 0.001, ****p < 0.0001. All grouped analyses had a power > 0.99.

Figure 2A,B shows that the viral load was significantly higher in the time period in which infections were predominantly caused by the Delta variant (median CT 18 or 6.55 log_10_ c/mL, Table 1), when compared to the non-VOC or Alpha periods (median CT 20 or 5.98 log_10_ c/mL).

**Table 1:** comparison of CT values and viral loads between non-VOC, Alpha and Delta variants of SARS-CoV-2.

	N	CT value			Viral Load (log10 copies/mL)		
		Median	Mean	95% CI	Median	Mean	95% CI
**Time periods**							
Non-VOC period	8630	20	22.31	22.17–22.45	5.98	5.38	5.34–5.42
Alpha period	7177	20	22.26	22.10–22.43	5.98	5.41	5.36–5.45
Delta period	378	18	20.28	19.58–20.98	6.55	5.98	5.80–6.16
**WGS confirmed variants**							
Non-VOC	631	19	19.48	19.14–19.81	6.26	6.13	6.03–6.22
Alpha	1240	18	18.81	18.58–19.04	6.55	6.32	6.25–6.38
Delta	87	16	16.71	15.88–17.54	7.11	6.91	6.68–7.14

### Viral load comparison of sequence-confirmed variants

Since the tested SARS-CoV-2 variants in the defined time periods for non-VOC, Alpha and Delta are based on assumption and will also include a small portion of different variants, we aimed to confirm these findings by comparing viral loads of only sequence-confirmed variants, of which the great majority was randomly selected and determined as part of the regional and national surveillance program. To this end, viral loads of a subset of WGS-confirmed non-VOC (n = 631), Alpha (n = 1240) and Delta (n = 87) variants were compared (Table 1, Fig. 2C,D). These results confirmed that there was an overall higher viral load in the Delta variant group (median CT 16 or 7.11 log_10_ c/mL, Table 1) when compared to the non-VOC (median CT 19 or 6.26 log_10_ c/mL, Table 1) or Alpha variant (median CT 18 or 6.55 log_10_ c/mL, Table 1). Additionally, the viral load of the Alpha variant group was increased compared to the non-VOC group.

### Viral load comparison between age groups and sexes

**Figure 3:**
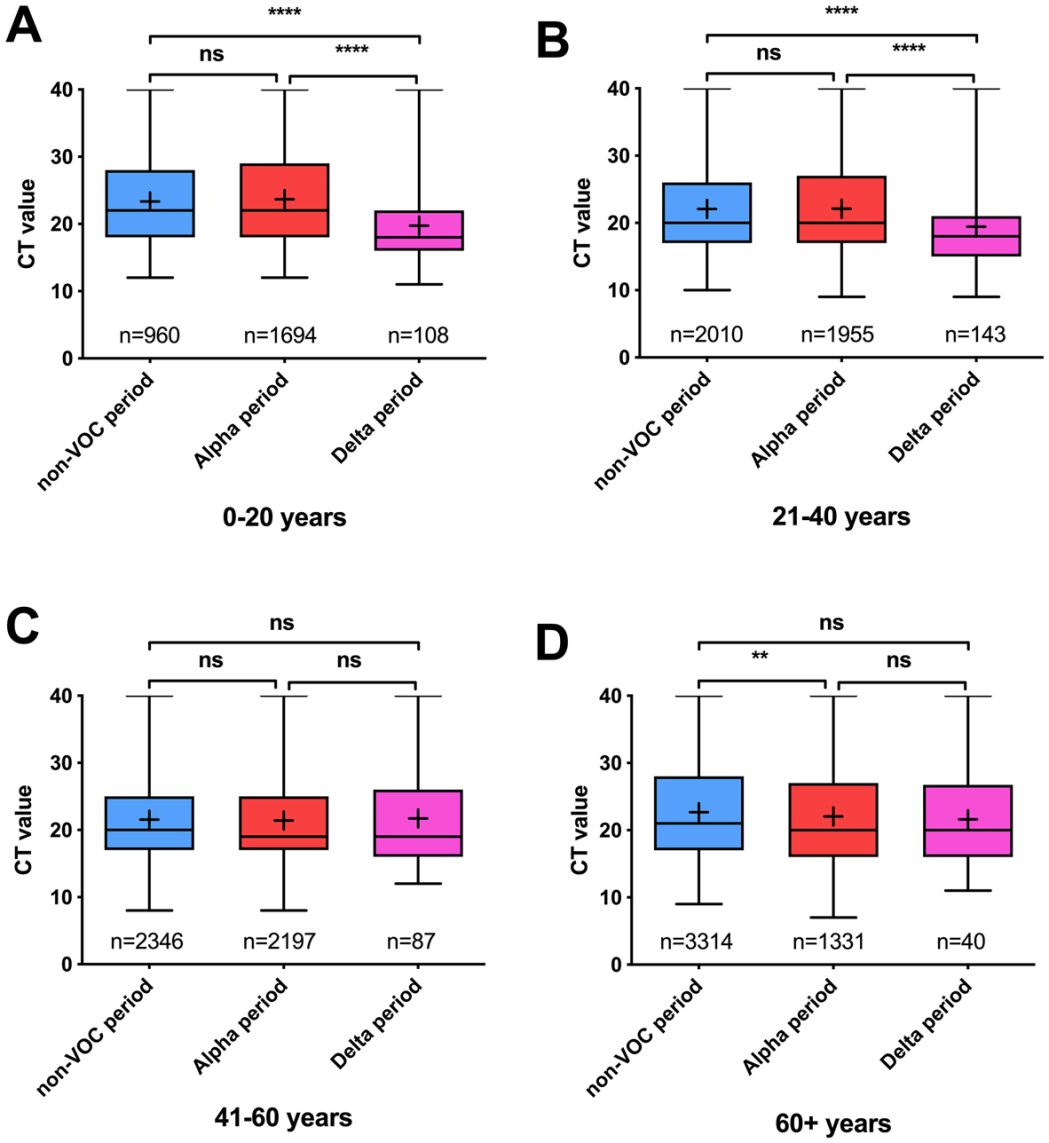
CT values per time period dominated by non-VOC (Dec 2020–Feb 2020), Alpha (Mar 2020–Jun 2020) and Delta (Jul 2020) SARS-CoV-2 variants for age groups 0–20 (**A**), 21–40 (**B**), 41–60 (**C**) and 60+ years (**D**). *Ns* not significant, **p < 0.01, ***P < 0.001, ****p < 0.0001.

Because the average virus load could also be different due to a difference in age of the tested population, we compared the viral loads of samples tested in the non-VOC, Alpha and Delta time periods in 4 different age groups (Fig. 3, Table 2). While the increase in viral load of the Delta variant was respectively 14-fold and fourfold higher in age groups 0–20 and 21–40 years, no difference was present in age groups 41–60 and 61+ years (Fig. 3, Table 2). This trend in age groups was inversely related to the amount of fully vaccinated individuals in these age groups in the same geographical area (Fig. S3). The mean age for samples collected during the non-VOC, Alpha or Delta period within each age group was highly comparable as shown in Table S2.

**Table 2:** Comparison of CT values and viral loads between non-VOC, Alpha and Delta variants of SARS-CoV-2 per age group.

	N	CT value			Viral Load (log10 copies/mL)		
		Median	Mean	95% CI	Median	Mean	95% CI
**Age group 0–20 years**							
Non-VOC period	960	22	23.35	22.93–23.76	5.41	5.08	4.97–5.20
Alpha period	1694	22	23.70	23.36–24.04	5.41	5.02	4.93–5.11
Delta period	108	18	19.75	18.52–20.98	6.55	6.11	5.80–6.43
**Age group 21–40 years**							
Non-VOC period	2010	20	22.09	21.81–22.38	5.98	5.44	5.36–5.52
Alpha period	1955	20	22.12	21.81–22.43	5.98	5.44	5.36–5.52
Delta period	143	18	19.44	18.36–20.52	6.55	6.19	5.91–6.47
**Age group 41–60 years**							
Non-VOC period	2346	20	21.55	21.29–21.81	5.98	5.58	5.51–5.65
Alpha period	2197	19	21.42	21.14–21.70	6.26	5.63	5.56–5.71
Delta period	87	19	21.71	20.08–23.35	6.26	5.60	5.19–6.02
**Age group 61+ years**							
Non-VOC period	3314	21	22.69	22.45–22.93	5.70	5.28	5.22–5.35
Alpha period	1331	20	22.05	21.66–22.43	5.98	5.47	5.37–5.57
Delta period	40	20	21.60	19.12–24.08	5.98	5.65	5.04–6.27

In addition, the viral load in the total dataset and different age groups was stratified by sex. In agreement with the previous results for the general population, males and females both harbored significantly higher loads in samples from the Delta period compared to the non-VOC and Alpha periods in age groups 0–20 and 21–40 years, but not in age groups 41–60 years or 61+ years (Fig. S4, Table S3). The only difference was that the viral load in samples from the Alpha period was significantly higher compared to samples from the non-VOC period in age group 61+ for females but not males.

## Discussion

Recent studies have indicated that the Delta variant is associated with increased viral loads, implying enhanced infectivity of this variant^3–5^. Nevertheless, the limited size of datasets used in these studies as well as the different diagnostic methods could lead to bias and preliminary conclusions with regard to the viral load and infectivity that is associated with the Delta variant. In this study we have compared the CT-values and viral load obtained from 16,185 samples that were tested positive during the time periods in which the non-VOC, Alpha and Delta variants were dominant, as evidenced by genomic surveillance of SARS-CoV-2 in the South Limburg region of The Netherlands. We established that samples that were tested positive since the Delta variant became dominant contained significantly higher loads of SARS-CoV-2 compared to samples that were tested during periods in which the non-VOC or Alpha variant were dominant. Based on the standard curve used for conversion of CT values to viral loads (Fig. S1A), it is estimated that samples harboring the Delta variant contain about fourfold higher loads of SARS-CoV-2 compared to samples harboring non-VOC or the Alpha variant. These observations were confirmed using a subset of WGS-confirmed samples in which samples containing the Delta variant harbored significantly higher loads of SARS-CoV-2 compared to their non-VOC (about sevenfold) or Alpha variant (about fourfold) counterparts. Interestingly, when using WGS-confirmed data, which consists of samples < CT30, a significantly higher load was observed for samples harboring the Alpha variant compared to the non-VOC variant. When performing the same analysis by only including samples < CT30 for non-WGS confirmed samples, there was also a moderate increased viral load observed for samples obtained during the period during which the Alpha variant was dominant versus samples collected during the non-VOC period (Fig. S2). The proportion of samples per patient category (public health service, nursing homes, general practitioner and commercial) as well as median age and the ratio of male/female were very similar when comparing the total and WGS-confirmed datasets (Table S1). By analyzing the viral loads in different age groups, we found that the increase in viral load of the Delta variant was only present in the younger population (age groups 0–20 and 21–40), regardless of sex, but not in age groups of 40 and up. Since the vaccination degree in our region was considerably higher in people older than 40, this may suggest that fully vaccinated individuals, on average, harbor lower viral loads compared to unvaccinated individuals as was previously also reported by Levine-Tiefenbrun et al.^9^ within the first 2 months after vaccination. The amount of fully vaccinated individuals in the South-Limburg region only starting accumulating rapidly in week 20, 5 weeks before the transition of the Alpha to the Delta period^10^. Therefore, the potential effect of vaccination on viral load reduction would likely be greater for samples obtained from individuals who became infected during the Delta compared to the Alpha or non-VOC period. Also, the proportion of vaccine breakthrough infections was reported to be higher for people infected with the Delta variant compared to Alpha or non-VOC^10–12^. Recently, it was reported that samples from people who are infected with the Delta variant harbor up to 1000 times higher viral loads than people infected with the clade 19A/19B viruses^5^. We did not observe such differences in viral load compared to the non-VOC or Alpha variant. Nevertheless, it has to be noted that the former study^5^ comprised a much smaller dataset (62 samples harboring Delta variant vs 63 samples harboring clade 19A/19B isolates) which was part of an outbreak and compared the Delta variant to a mixture of samples harboring nextstrain clade 19A/19B variants, which did not harbor the D614G mutation that enhances infectivity^13–15^ and were much closer related to the original Wuhan strain than the non-VOC isolates in our study. In agreement with our study, King et al*.*^16^ also reported a more subtle increase in viral load for saliva samples harboring the Delta variant since the median CT value was about 1.3 values lower compared to Alpha and about three CT values lower compared to non-VOC samples. This study also observed a significant increase in viral load when comparing Alpha to non-VOC, which was only observed in the WGS dataset in our study. Several studies have suggested that the increase in viral load for the Delta variant compared to previous variants could reflect an enhanced replication rate for this variant in the airways of infected individuals^16,17^. Indeed, in vitro infection experiments in both cell culture and organoid models have shown that the Delta variant has a significantly higher replication rate compared to the Alpha and the wild-type variant harboring the D614G mutation^18,19^. The increased viral load in respiratory samples harboring the Delta variant could also induce the generation of viral aerosols since an analytic study performed by Lee^17^ has demonstrated that higher viral loads in respiratory fluids decrease the minimum size of virus-laden respiratory particles. The latter study has also shown that the minimum viral load required in respiratory fluids to generate viral aerosols is about 10^6^ viral copies/mL, a requirement that is fulfilled in a greater proportion of samples harboring the Delta variant compared to samples harboring Alpha or non-VOC in our study.

One of the strengths of this study is the large dataset of samples. Furthermore, follow-up samples obtained from the same subject were filtered out of the dataset, to avoid bias. Finally, extensive genomic surveillance in the region allowed us to accurately estimate the dominant period for each variant as WGS of a subset of samples confirmed the observed trends. Limitations of this study can be found in the fact that the available dataset of the Delta variant is still limited in size compared to the other datasets and the fact that the non-VOC group consists of many different pangolin lineages. In addition, no information about the vaccination status or disease severity at the individual level was obtained, so the effect of vaccination or symptom development on viral loads could not be determined in this study.

## Conclusion

In summary, our study shows that samples from individuals that are infected with the Delta variant harbor about fourfold higher loads of SARS-CoV-2 compared to individuals that are infected with non-VOC or the Alpha variant, which is significantly lower than previously reported^5^.

## Methods

### Study population

In this study, nasopharyngeal samples that were tested positive for SARS-CoV-2 (total n = 16,185) were collected from 1 December 2020 until 24 July 2021 by the South Limburg Public Health Service (n = 13,927), nursing homes (n = 1932), general practitioners (n = 226) and commercial parties (n = 100). Samples from hospital patients and personnel were excluded as they were regularly tested and could introduce bias in the dataset. All nasopharyngeal swabs were immediately deposited in 3 mL GLY viral transport medium (Mediaproducts BV) after collection and stored under the same conditions prior to testing. The vast majority of samples was collected by the South Limburg Public Health Service. Their testing policy was aimed at testing symptomatic individuals and contacts of positive individuals via contact tracing and this strategy was not changed over the course of this study. This means that both symptomatic and asymptomatic individuals were included in this study. As the testing policy for adults and children was not changed over the course of this study, nasal swabs were collected from children throughout all time points during this study.

### Laboratory investigation and determination of viral load

RNA extraction was performed mainly by using the chemagic Viral DNA/RNA 300 Kit H96 kit (Perkin Elmer) according to the manufacturer’s instructions and was eluted in 100 μL elution buffer. A small fraction (6%) of extractions were performed by using the MagNA Pure 96 DNA and Viral NA Small Volume Kit (Roche Diagnostics) according to the manufacturer’s instructions and was eluted in 100 μL (50 μL elution buffer + 50 μL water). Both extraction systems were found to yield highly comparable RNA concentrations, leading to comparable viral loads and limits of detection (2 log10 viral copies/mL; see Fig. S1). All RT-qPCR data was produced on Quantstudio 5 systems (Thermofisher). Amplification was performed by multiplex PCR, using the E gene target^20^ in addition to the N1 target^21^ and mCMV-ie as internal control. Concentrations for the multiplex PCR were 400 nM primer and 200 nM probe for both the E and N1 targets and 300 nM primer (sense: 5’-CAACATTGACCACGCACTAGATG-3’; antisense: 5’-TTAAACTCCCCAGGCAATGAA-3’) and 200 nM probe (5’-TCTTGGCCCATGCGGCACG-3’) for the internal control. The assay consisted of 5 µl of TaqPath 1-Step RT-qPCR Master Mix (Thermofisher), 10 µl RNA eluate and 5 µl of primer/probe mix. Cycling conditions were 2 min at 25 °C, 15 min at 50 °C, 2 min at 95 °C followed by 42 cycles of 3 s at 95 °C and 30 s at 60 °C. Viral loads were obtained using a standard curve in which the CT value was plotted in function of serial dilutions of a quantified viral stock solution (Fig. S1A). All positive results below 2 log10 copies/mL virus were transformed to 2 log10 copies/mL since these loads were outside of the linear range of the PCR.

### Next-generation sequencing

Sequencing was performed using the PCR tiling of SARS-CoV-2 virus with Native Barcoding Expansion 96 (EXP-NBD196) protocol (Version: PTCN_9103_v109_revH_13Jul2020) of Oxford Nanopore technologies, with minor modifications and using the primers previously published by Oude Munnink et al.^22^ Briefly, the only modifications were extending the barcode and adaptor ligation steps up to 60 min and loading 48 samples per flow cell. Bioinformatic analysis was performed using an in-house developed pipeline MACOVID (https://github.com/MUMC-MEDMIC/MACOVID) that is based on Artic v1.1.3.

### Statistics

The analysis of data was performed using Graphpad Prism version 5.04 via the Kruskal–Wallis and post-hoc Dunn’s Multiple Comparison tests. P < 0.05 was considered to be statistically significant.

Power analysis was performed using R statistical software, version 3.6.2 (R Foundation for Statistical Computing, Vienna, Austria). The a priori calculated sample size to detect small differences (Cohen’s f value 0.1) was 322 samples per group to reach a power of 0.8, whereas the calculated sample size to detect medium differences (Cohen’s f value 0.25) was 52 samples per group to reach a power of 0.8.

### Ethical statement

The Medical Review Ethics Committee of the Maastricht UMC+ confirmed that the Medical Research Involving Human Subjects Act (WMO) does not apply to the above mentioned study and that neither an official approval of this study by the committee nor informed consent from the participants is required (METC reference number 2021-2838).

### Accordance statement

The authors confirm that all experiments were performed in accordance with local guidelines and regulations.

## Supplementary Information

Supplementary Information.

## Data Availability

The datasets used in this study are available from the corresponding author upon reasonable request.
