# Supplementary material for: Infections with the SARS-CoV-2 Delta variant exhibit fourfold increased viral loads in the upper airways compared to Alpha or non-variants of concern
Source: Sci Rep. 2022 Aug 17;12:13922. doi: 10.1038/s41598-022-18279-5 (PMC9382600; doi:10.1038/s41598-022-18279-5)
Supplement: Supplementary file 1 — Supplementary Information. [file 41598_2022_18279_MOESM1_ESM.pdf]

Supplementary material

Infections with the SARS-CoV-2 Delta variant exhibit 4-fold increased viral loads in the upper airways compared to Alpha or non-Variants of Concern

Christian J.H. von Wintersdorff[1]*, Jozef Dingemans[1]*, Lieke B. van Alphen[1], Petra F.G. Wolffs[1], Brian M.J.W. van der Veer[1], Christian J.P.A. Hoebe[2], Paul H.M. Savelkoul[1]

A

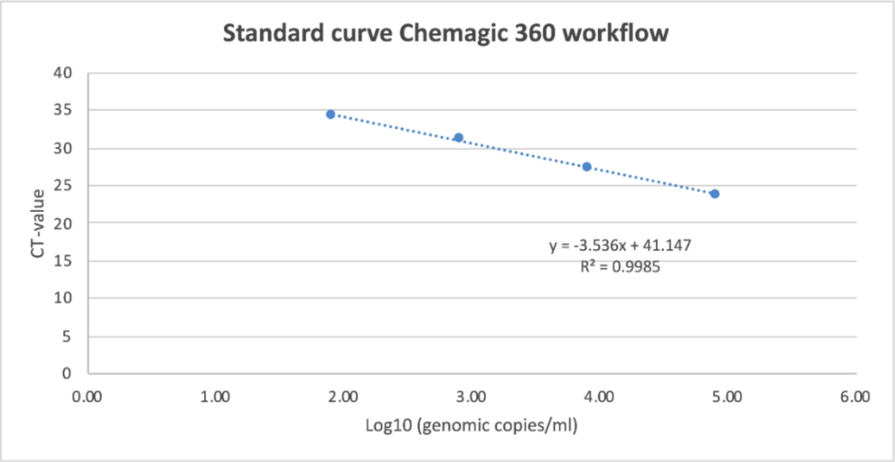

B

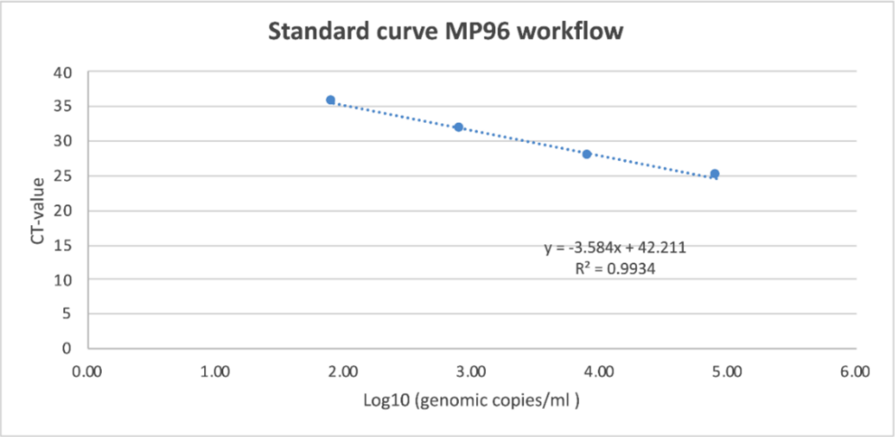

Figure S1. A. Standard curve of the CT value in function of log10 genomic copies/ml for RNA extraction using the Chemagic 360 (Perkin-Elmer). **B.** Standard curve of the CT value in function of log10 genomic copies/ml for RNA extraction using the MagnaPure 96 (Roche). The standard curve was based on 4 measurements per sample with a known viral load that was tested. The equation for the Chemagic 360 workflow was used to convert CT values to viral loads as the majority of samples were tested using this workflow.

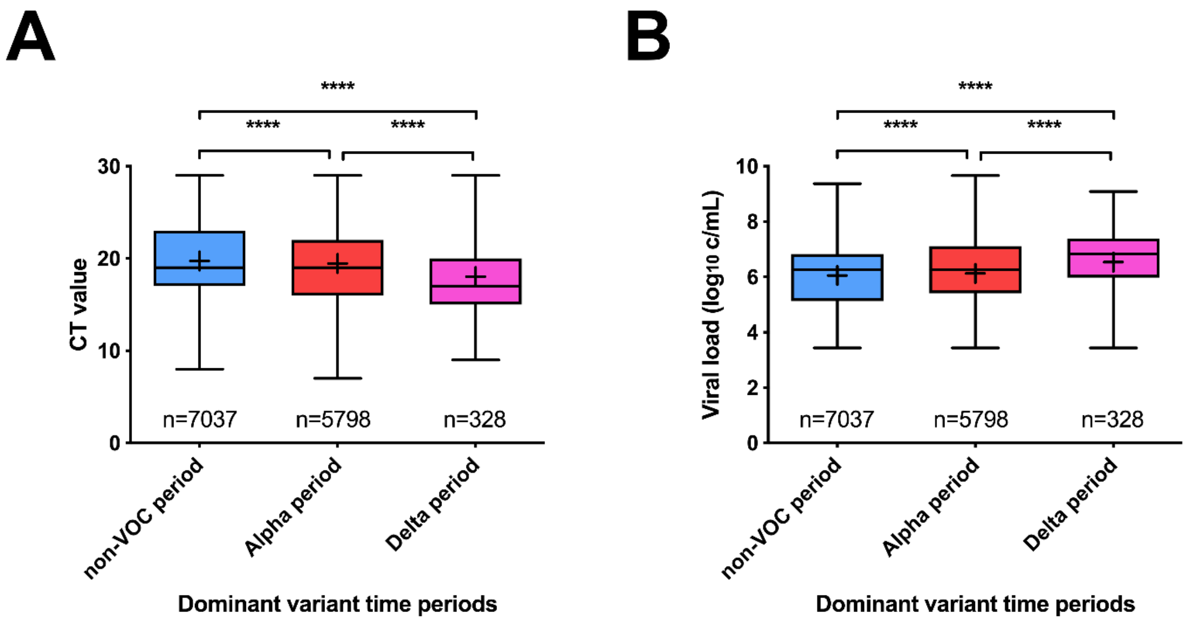

Figure S2. CT values (A) and viral loads (B) per time period dominated by non-VOC (Dec 2020 – Feb 2020), Alpha (Mar 2020 – Jun 2020) and Delta (Jul 2020) SARS-CoV-2 variants for samples <CT30. Ns: not significant, ****: $p < 0.0001$.

Age	Vaccination started	Vaccination completed
<15	7%	0%
15-19	52%	15%
20-24	50%	24%
25-29	52%	35%
30-34	58%	43%
35-39	65%	52%
40-44	70%	59%
45-49	69%	58%
50-54	74%	64%
55-59	81%	69%
60-64	94%	80%
65-69	88%	74%
70-74	89%	69%
75-79	91%	72%
80+	85%	67%
Total	66%	50%

Figure S3. SARS-CoV-2 vaccination status per age category of South Limburg, The Netherlands on August 9 of 2021

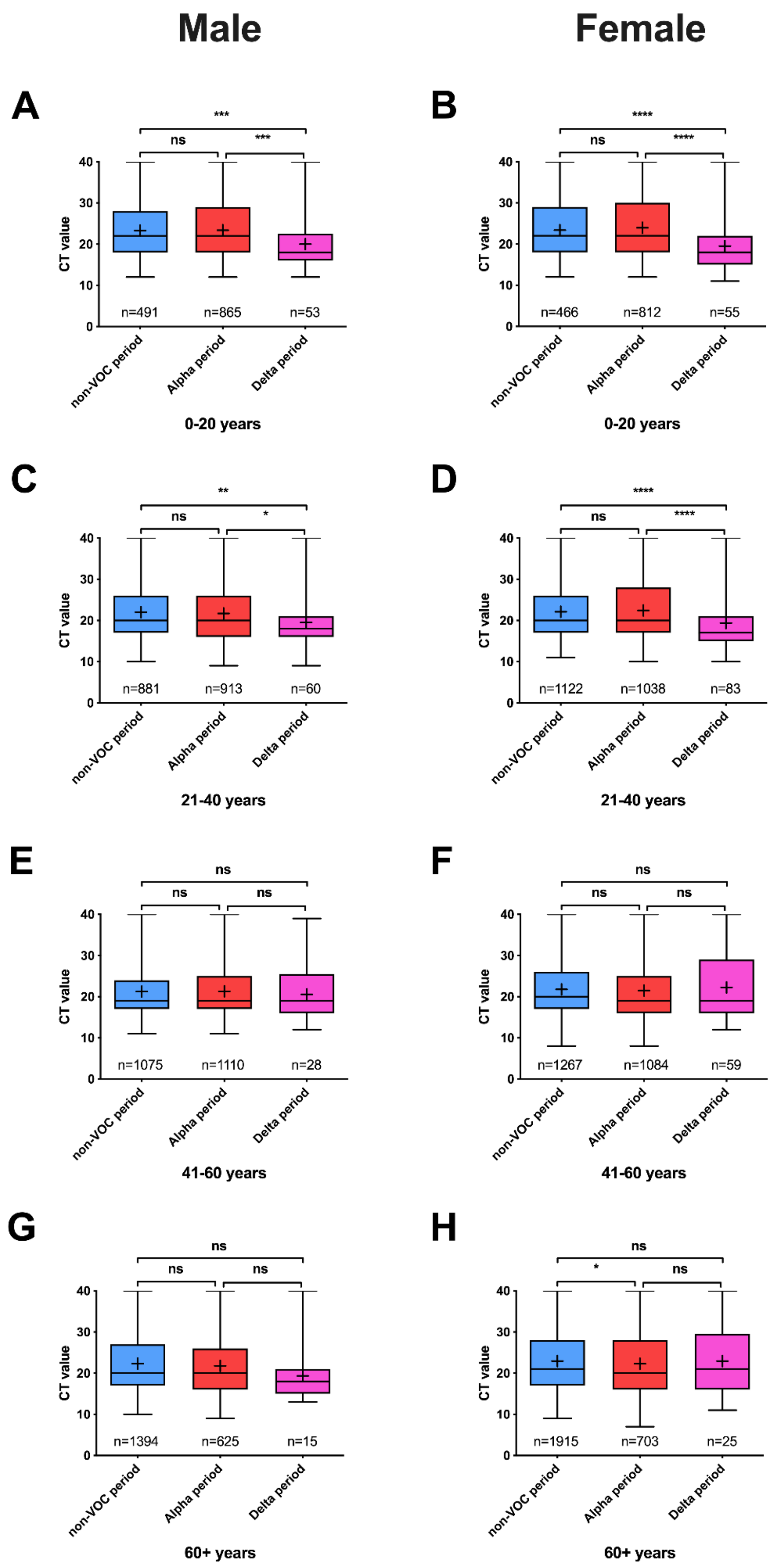

Figure S4. CT values stratified by sex for age groups 0-20 (A-B), 21-40 (C-D), 41-60 (E-F) and 60+ years (G-H). Ns: not significant, *: p < 0.05, **: p < 0.01, ***: P < 0.001, ****: p < 0.0001.

Table S1. Population characteristics of total vs WGS dataset

	Total (n=16,185)	WGS-confirmed (n=1,958)
**South Limburg Public Health Service**	13,927 (86%)	1,773 (90.6%)
**Nursing homes**	1,932 (12%)	134 (6.8%)
**General practitioners**	226 (1.4%)	46 (2.3%)
**Commercial parties**	100 (0.6%)	5 (0.3%)
**Median Age (years)**	47	46
**Male**	7,510 (46%)	912 (47%)
**Female**	8,629 (53%)	1,035 (53%)

Table S2. Mean and median age per age group.

	N	Mean age (years)
Age group 0-20 years		
Non-VOC period	960	14.32
Alpha period	1694	12.63
Delta period	108	14.52
Age group 21-40 years		
Non-VOC period	2010	29.97
Alpha period	1955	30.21
Delta period	143	27.64
Age group 41-60 years		
Non-VOC period	2346	51.40
Alpha period	2197	50.77
Delta period	87	49.67
Age group 61+ years		
Non-VOC period	3314	77.00
Alpha period	1331	72.70
Delta period	40	79.93

Table S3. Comparison of CT values and viral loads for non-VOC, Alpha and Delta variants of SARS-CoV-2 between males and females.

Male							
		CT value			Viral Load (log10 copies/mL)		
	N	Median	Mean	95% CI	Median	Mean	95% CI
Total data set							
Non-VOC period	3840	20	22.09	21.88-22.29	5.98	5.44	5.38-5.49
Alpha period	3513	20	22.01	21.78-22.23	5.98	5.47	5.41-5.53
Delta period	156	18	19.85	18.81-20.88	6.55	6.10	5.83-6.36
Age group 0-20 years							
Non-VOC period	491	22	23.31	22.72-23.89	5.41	5.10	4.95-5.26
Alpha period	865	22	23.37	22.90-23.84	5.41	5.10	4.98-5.23
Delta period	53	18	20.00	18.09-21.91	6.55	6.07	5.59-6.55
Age group 21-40 years							
Non-VOC period	881	20	22.02	21.59-22.46	5.98	5.46	5.34-5.57
Alpha period	913	20	21.71	21.27-22.14	5.98	5.54	5.43-5.66
Delta period	60	18	19.50	17.92-21.08	6.55	6.18	5.78-6.58
Age group 41-60 years							
Non-VOC period	1075	19	21.26	20.88-21.63	6.26	5.66	5.56-5.76
Alpha period	1120	19	21.32	20.94-21.70	6.26	5.66	5.55-5.76
Delta period	28	19	20.57	17.88-23.27	6.26	5.87	5.15-6.58
Age group 61+ years							
Non-VOC period	1394	20	22.33	21.97-22.68	5.98	5.37	5.28-5.47
Alpha period	625	20	21.80	21.25-22.34	5.98	5.53	5.39-5.68
Delta period	15	18	19.33	15.67-23.00	6.55	6.28	5.44-7.12

Female							
		CT value			Viral Load (log10 copies/mL)		
	N	Median	Mean	95% CI	Median	Mean	95% CI
Total data set							
Non-VOC period	4770	21	22.49	22.30-22.69	5.70	5.33	5.28-5.38
Alpha period	3637	20	22.47	22.24-22.71	5.98	5.36	5.30-5.42
Delta period	222	18	20.59	19.63-21.54	6.55	5.89	5.65-6.14
Age group 0-20 years							
Non-VOC period	466	22	23.38	22.78-23.97	5.41	5.06	4.90-5.23
Alpha period	812	22	23.94	23.44-24.43	5.41	4.96	4.83-5.09
Delta period	55	18	19.51	17.90-21.12	6.55	6.15	5.73-6.58
Age group 21-40 years							
Non-VOC period	1122	20	22.13	21.75-22.51	5.98	5.43	5.33-5.53
Alpha period	1038	20	22.46	22.02-22.90	5.98	5.36	5.25-5.48
Delta period	83	17	19.40	17.90-20.90	6.83	6.20	5.80-6.60
Age group 41-60 years							
Non-VOC period	1267	20	21.79	21.43-22.14	5.98	5.52	5.42-5.61
Alpha period	1084	19	21.51	21.10-21.92	6.26	5.61	5.51-5.72
Delta period	59	19	22.25	20.17-24.34	6.26	5.48	4.95-6.00
Age group 61+ years							
Non-VOC period	1915	21	22.96	22.64-23.29	5.70	5.22	5.13-5.30
Alpha period	703	20	22.28	21.73-22.83	5.98	5.41	5.27-5.55
Delta period	25	21	22.96	19.58-26.34	5.70	5.28	4.43-6.13
